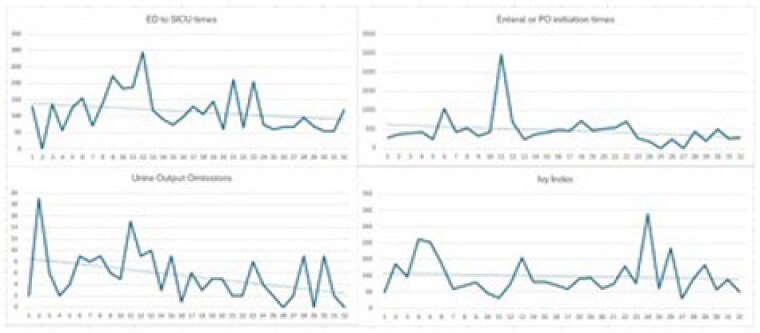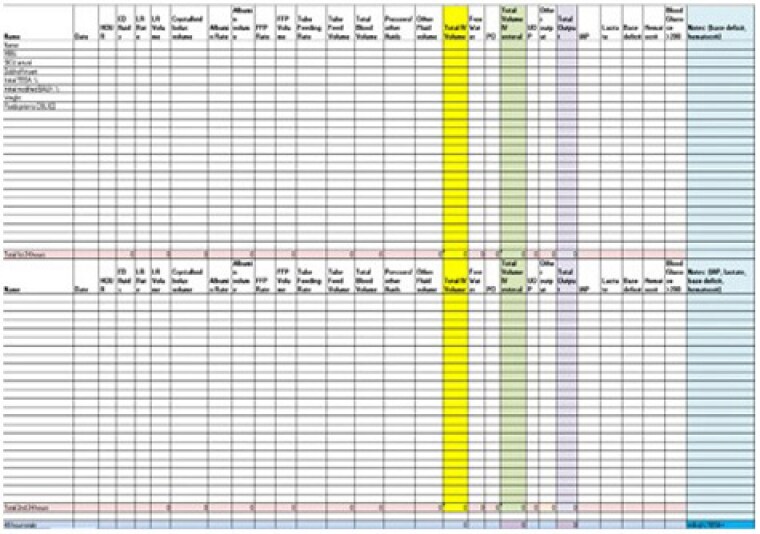# 670 Multidisciplinary Debrief Post Fluid Resuscitation

**DOI:** 10.1093/jbcr/iraf019.299

**Published:** 2025-04-01

**Authors:** John Loftus, Nicole Bernal, Lauren Harrison, Beth McGuire, Nidhi Aravapalli, Michael Young, Laura Pezzopane, Ariel Rodgers

**Affiliations:** The Ohio State Comprehensive Burn Center; The Ohio State University, Wexner Medical Center; The Ohio State University, Wexner Medical Center; The Ohio State University; The Ohio State University; The Ohio State University, Wexner Medical Center; The Ohio State University, Wexner Medical Center; The Ohio State University, Wexner Medical Center

## Abstract

**Introduction:**

Fluid Resuscitation is carried out in a surgical intensive care unit (SICU) separate from the acute care burn unit. A provider committee was developed to review a summary of the first 48 hours of a patient’s fluid resuscitation and provide feedback. A multidisciplinary team debrief was initiated to discuss the committee findings, review the fluid resuscitation clinical practice guideline (FRCPG) and help foster the relationship/open communication between the critical care and burn teams.

**Methods:**

The Lund Browder calculation was used and the FRCPG was initiated on patients who presented to the hospital with >20% TBSA or 15%-19% TBSA with additional risk factors for dehydration. The Brooke formula was used to determine the IV fluid starting rate.

A spreadsheet (Fig. 1), consisting of, but not limited to, hourly urine output and fluid rate, enteral access time, and SICU arrival time from the emergency department (ED) is completed by a member of the Burn QI committee and forwarded to the provider committee within 24-48 hours of completion of the fluid resuscitation. The committee reviews the spreadsheet to determine opportunities for improvement, call outs for successes and FRCPG compliance. The debrief occurs within two weeks of the fluid resuscitation completion for review of the committee’s feedback and further review of the FRCPG to answer any questions from the bedside team.

**Results:**

Primary result was a better understanding of the FRCPG by the residents and the nursing staff, therefore increasing compliance. A non-measurable result of the debriefs was a team that was comfortable discussing the fluid resuscitation in real time due to a better understanding of how/why the FRCPG impacts a patient’s outcomes. Secondary result was addressing consistent barriers found during the provider reviews and discussing with the team how to impact those delays/compliance. The use of the spreadsheet gave transparency to the indicators for under and over-resuscitation including variances from the expected documentation of hourly urine outputs (UOP), IV fluid titration rates, and the need to get the patients to SICU from ED as quickly as possible.

**Conclusions:**

Over a twelve month period, we have conducted debriefs for 32 fluid resuscitation cases. We have seen impactful improvements with all four of our data points (Fig. 2): Ivy Index, SICU arrival time, enteral access, hourly UOP documentation. In addition, we have also been able to reap the benefits of a stronger, more cohesive team that works together to improve the outcomes of our larger burned patients.

**Applicability of Research to Practice:**

A multidisciplinary team resuscitation debrief creates an educational forum allowing a platform to identify ways to improve the resuscitation process of guideline compliance and team communication/understanding.

**Funding for the Study:**

N/A